# Transcriptome Analysis of Post-Hatch Breast Muscle in Legacy and Modern Broiler Chickens Reveals Enrichment of Several Regulators of Myogenic Growth

**DOI:** 10.1371/journal.pone.0122525

**Published:** 2015-03-30

**Authors:** Richard V. N. Davis, Susan J. Lamont, Max F. Rothschild, Michael E. Persia, Chris M. Ashwell, Carl J. Schmidt

**Affiliations:** 1 Dept. Biological Sciences, University of Delaware, Newark, Delaware, 19716, United States of America; 2 Dept. of Animal Science, Iowa State University, Ames, Iowa, 50011, United States of America; 3 Dept. of Poultry Science, North Carolina State University, Raleigh, North Carolina, 27695, United States of America; 4 Dept. of Animal and Food Sciences, University of Delaware, Newark, Delaware, 19716, United States of America; University of Massachusetts Medical, UNITED STATES

## Abstract

Agriculture provides excellent model systems for understanding how selective pressure, as applied by humans, can affect the genomes of plants and animals. One such system is modern poultry breeding in which intensive genetic selection has been applied for meat production in the domesticated chicken. As a result, modern meat-type chickens (broilers) exhibit enhanced growth, especially of the skeletal muscle, relative to their legacy counterparts. Comparative studies of modern and legacy broiler chickens provide an opportunity to identify genes and pathways affected by this human-directed evolution. This study used RNA-seq to compare the transcriptomes of a modern and a legacy broiler line to identify differentially enriched genes in the breast muscle at days 6 and 21 post-hatch. Among the 15,945 genes analyzed, 10,841 were expressed at greater than 0.1 RPKM. At day 6 post-hatch 189 genes, including several regulators of myogenic growth and development, were differentially enriched between the two lines. The transcriptional profiles between lines at day 21 post-hatch identify 193 genes differentially enriched and still include genes associated with myogenic growth. This study identified differentially enriched genes that regulate myogenic growth and differentiation between the modern and legacy broiler lines. Specifically, differences in the ratios of several positive (IGF1, IGF1R, WFIKKN2) and negative (MSTN, ACE) myogenic growth regulators may help explain the differences underlying the enhanced growth characteristics of the modern broilers.

## Background

The advent of modern agriculture in the early 20^th^ century led to intensive genetic selection for meat (broiler) and egg (layer) production traits in the domesticated chicken. To improve production, the broiler chicken underwent genetic selection for rapid growth, particularly of the skeletal muscle tissue. In 1950 a broiler took 16 weeks before going to market while by 1990 birds were going to market by 6 to 7 weeks [1–4]. Because of this intense selective pressure, broiler chickens provide an excellent platform to identify the genes and alleles selected by this human-directed evolution.

One way to evaluate the effects of selection is to compare the growth characteristics and gene expression patterns of modern broilers with legacy birds that exhibit growth properties similar to those of meat-type birds prior to intense selection [5–8]. The legacy line used in this study is maintained at the University of Illinois as an inbred line with growth characteristics similar to those of meat-production birds from the late 1940s (we refer to these birds as Illinois birds). The Illinois birds are the result of a cross between New Hampshire males, and females carrying the Columbian feather pattern [8]. Our prior studies have focused on allometric, morphometric and feed efficiency comparisons between the modern Ross 708 broiler and the Illinois line [8]. At all time-points after D7 post-hatch until the end of the trial at D35 post-hatch, Ross 708 birds were significantly larger than the Illinois birds with the Ross 708 birds, on average depositing mass 1.8 times faster than the Illinois birds. This difference in mass was even more pronounced when only the breast muscle mass was compared. The Ross 708 birds deposited breast muscle mass at a rate 3.8 times greater than the Illinois birds [8].

Comparing the breast muscle growth patterns between the two lines also revealed a significant difference following day 14 post-hatch. In the Illinois birds, the normalized breast muscle mass (breast muscle mass/bird mass) remained constant after day 14 whereas the Ross 708 normalized breast muscle mass continued to increase [8]. Given these observations, we hypothesized that genes affecting muscle growth and differentiation along with ones affecting energy metabolism would be differentially regulated between the breast muscle of the Ross 708 and Illinois lines, and that the transcriptomes would have different relationships before and after the growth-inflection point of day 14. To test this hypothesis, RNA-seq was used to compare the gene expression patterns of the breast muscle from Ross 708 and Illinois birds bracketing the 14-day post-hatch period. The transcript levels of 15,945 genes were analyzed in the breast muscle of post-hatch day 6 (D6) and day 21 (D21) Illinois and Ross 708 chickens.

## Methods

### Animal Rearing and Tissue Collection

Ross 708 and Illinois chickens were raised in the same house on the University of Delaware farm and provided with food and water *ad libitum*. Birds were euthanized by cervical dislocation and tissue samples were excised from the posterior region of the left *pectoralis major* muscle, flash frozen in liquid nitrogen and then stored at -80 ^o^C until RNA isolation.

### Ethics Statement

This study was carried out in strict accordance with the recommendations in the Guide for the Care and Use of Laboratory Animals of the National Institutes of Health. The protocol was approved by the Committee on the Ethics of Animal Experiments of the University of Delaware (Permit Number: 2703-12-10).

### RNA Isolation

Total RNA was isolated from approximately 30 mg samples of *Pectoralis major* tissue using the Qiagen RNeasy Mini Kit as per the manufacturer’s instructions. Total RNA was extracted from 12 D6 samples (6 Ross 708, 6 Illinois) and 12 D21 samples (6 Ross 708, 6 Illinois). RNA concentration was assessed with a Nanodrop ND-100 spectrophotometer. Overall quality of the RNA was assessed using an Agilent 2100 Bioanalyzer.

### mRNA Purification, cDNA synthesis and RNA-seq Library Preparation

mRNA was purified from total RNA extracts by separation with magnetic oligo-dt beads. cDNA constructs were synthesized from purified mRNA samples and prepared for sequencing using the Ilumina TruSeq RNA Sample Preparation Kit (Ilumina, San Diego, CA) as per the manufacturers instructions.

### Sequencing and Alignment

Prepared libraries were sequenced using the HiSeq 2000 Sequencing System (Ilumina) at the Delaware Biotechnology Institute’s Sequencing and Genotyping Center (Newark, DE). All D6 libraries, both Ross 708 and Illinois, were sequenced to a depth of ~10 million reads per library. For the D21 time point, four Ross 708 libraries and two Illinois libraries were also sequenced at a depth of ~10 million reads per library. Due to improvements in efficiency at the sequencing facility two D21 Ross 708 libraries and three D21 Illinois libraries were sequenced at a depth of ~30 million reads per library. 12 D6 libraries (6 Ross 708 and 6 Illinois) and 11 D21 libraries (6 Ross 708 and 5 Illinois) were aligned to the 2006 version of the chicken genome sequence and annotation using the ERANGE package [9].

### Power Analysis and Experimental Design

A random subset of D6 samples (2 Ross 708 and 2 Illinois) was supplied to Scotty [10], a tool for power analysis and experimental design of RNA-seq experiments, in order to simulate pilot data, per the designer’s suggestion. The results of the power analysis indicate that the number of replicates and depth of sequencing provide sufficient power to detect >75% of differentially enriched genes at a p-value of 0.05. The same analysis was performed with the D21 samples and the results of the power analysis indicate that the number of replicates and depth of sequencing provide sufficient power to detect >75% of differentially enriched genes at a p-value of 0.05. All comparisons were made within time points and across lines, i.e. D6 Ross 708 is compared to D6 Illinois but is not directly compared to D21 Ross 708.

### Transcriptional Profiling and Analysis of Differential Gene Expression

The sequencing data discussed in this publication have been deposited in NCBI’s Gene Expression Omnibus [11] and are accessible through GEO series accession number GSE65217 (http://www.ncbi.nlm.nih.gov/geo/query/acc.cgi?acc=GSE65217). Statistical analyses were performed using JMP software. We determined the ratio of reads per kilobase of gene per million mapped reads (RPKM) between the Ross 708 and Illinois lines on D6 and D21 and performed a log_2_ transformation on this ratio. Genes whose ratios fell greater than 2 standard deviations from the mean, with a p-value < 0.05 were identified as differentially expressed between the two lines. Text mining was performed and informative terms (iTerms) were generated from the differentially expressed gene lists using eGIFT [12]. Gene ontology analysis was performed using the lists of differentially enriched genes with the AgBase GOSlim ontology, and the AgBase tools GORetriever and GOSlimViewer [13]. Any GO terms with >10 instances at either D6 or D21 were used to generate figures with R [14]. Finally, the 30 most enriched genes from each line at each time point were further examined by manual review of the literature.

### Quantitative reverse transcription polymerase chain reaction (qRT-PCR) Verification

Complimentary DNA (cDNA) was synthesized from previously isolated Ross 708 and Illinois breast muscle RNA using the Superscript II first strand synthesis kit (Invitrogen). cDNA concentration and quality were assessed with a Nanodrop ND-100 spectrophotometer (Nanodrop Technology, Wilmington, DE) and diluted to 90 ng/μl of cDNA. Diluted cDNA samples and 12 different primer pairs, designed to isolate specific genes, were used to conduct quantitative PCR. Quantitative PCR was done using the 7500 Fast Real Time PCR System (Applied Biosystems). The *glioblastoma amplified sequence* (GBAS) gene was used as the control gene to normalize for the amount of cDNA in the qPCR reaction. Each reaction was done in triplicate. Fold differences between the Heritage and Ross line gene expression levels were determined by delta-delta method, statistical analysis of Real Time PCR data [15].

Several myogenic regulatory genes were selected for verification via probe-based qPCR. Primers and probes were designed using Primer Express 3.0 software (Life Technologies) and checked for specific mapping to the chicken genome using BLAT [16]. Custom oligonucleotides were also designed for each primer-probe set to construct standard curves for quantification. These custom standards consisted of the forward primer, probe and reverse complement of the reverse primer each separated by a single nucleotide. Probe qPCR reactions were prepared using the QuantiTect probe qPCR kit (Qiagen) and reactions were performed using the 7500 Fast Real Time PCR System (Applied Biosystems).

## Results and Discussion

### Differential expression of breast muscle genes

**Fig 1:**
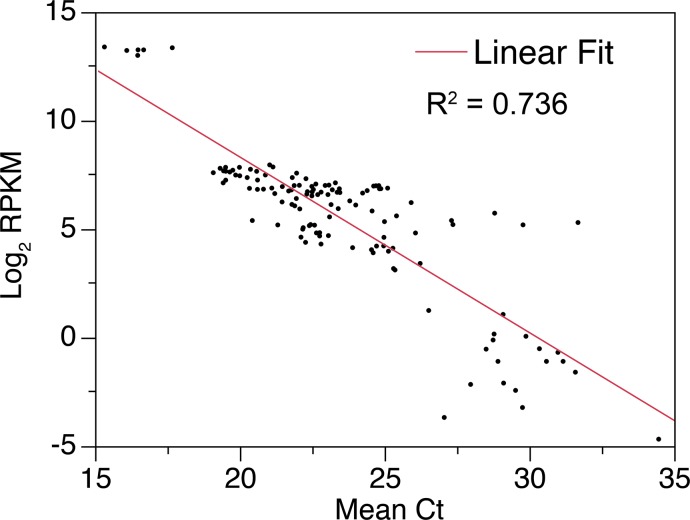
Comparison and correlation of the relative expression of selected genes as determined via RNA-seq (RPKM) and qRT-PCR. qPCR ct values are the mean of 3 replicates. ct values were determined using the delta-delta method. The GBAS (*glioblastoma amplified sequence*) gene was used to normalize the qPCR data for cDNA input.

**Fig 2:**
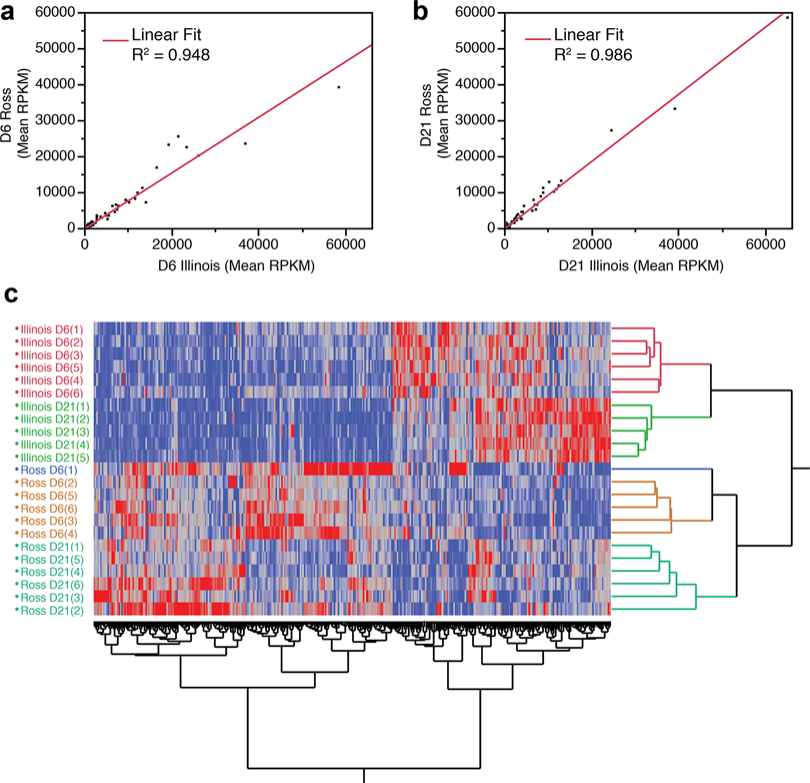
Scatter plot comparison of the expression level (in RPKM) of all genes between Ross 708 and Illinois lines on (A) post-hatch day 6 and (B) post-hatch day 21. (C) Hierarchical clustering of all significantly differentially expressed genes. (A&B) The expression levels of all 15,945 analyzed genes were compared for each time point in both lines, the line of best fit and R^2^ values are included. C) Hierarchical clustering was performed on 366 genes determined to be differentially enriched in at least one condition. Dendrograms are in a distance scale.

A total of 15,945 genes were analyzed for their expression level, expressed in reads per kilobase of transcript per million mapped reads (RPKM), in breast muscle tissue from both Ross 708 and Illinois birds; of these, 10,841 genes were expressed with an RPKM > 0.1, the threshold that corresponds to reliable results when assayed by quantitative RT-PCR (Fig 1). Comparing the RPKM values for all genes from the Ross and Illinois birds at day 6 and day 21 shows a high relationship between the samples (Day 6 R^2^ = 0.948 and Day 21 R^2^ = 0.986) (Fig 2A and 2B) and hierarchical clustering segregated the data first by line then by day (Fig 2C).

**Fig 3:**
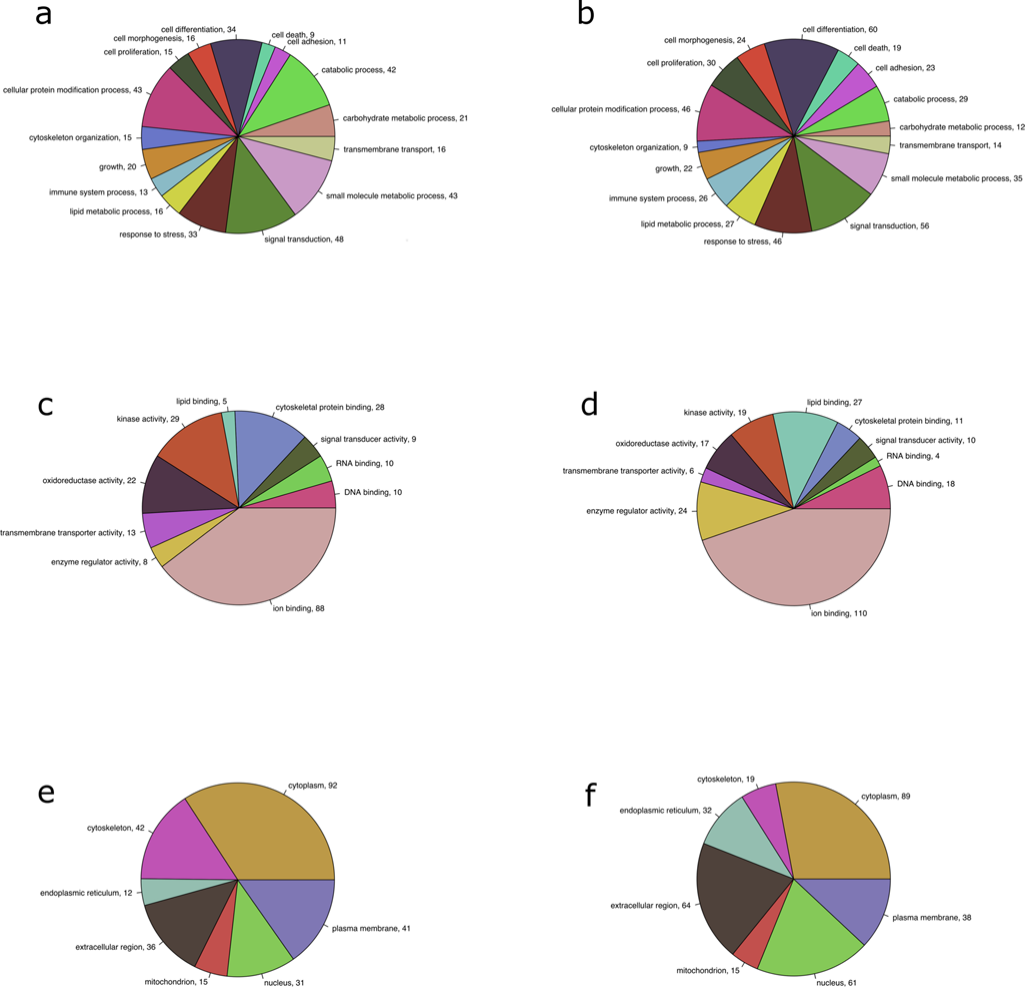
AgBase GOSlim terms for biological process (a & b), molecular function (c & d) and cellular component (e & f) ontologies generated from D6 and D21 significantly differentially enriched genes. All GOSlim terms with at least 10 instances at either D6 or D21 were included in the graphs for both time points. Chart labels are the GO term followed by the number of instances that term was generated by the differentially enriched gene lists.

Subsequently, genes were identified that exhibited significant differential enrichment between the two lines on either D6 or D21 post-hatch. The D6 Ross 708 breast muscle expressed 93 genes that were enriched relative to the Illinois samples, while the Illinois muscle showed enrichment for 96 gene transcripts (S1 Table). The D21 Ross 708 breast muscle was enriched for 138 transcripts while the Illinois muscle expressed 55 genes that were enriched relative to the Ross 708 D21 samples (S2 Table). Gene ontology (GO) analysis performed on the differentially enriched genes lists indicated that several important biological processes, including cell proliferation, cell differentiation, growth, catabolic processes, and metabolic processes, were differentially regulated at D6 (Fig 3a) and D21 (Fig 3b). Important molecular functions affected included lipid binding, oxidoreductase activity, kinase activity and other enzyme regulator activities (Fig 3c and 3d). Finally, differences in cellular components such as the extracellular region and the cytoskeleton were observed at both D6 (Fig 3e) and D21 (Fig 3f).

### Day 6 Differentially Enriched Genes

#### Muscle Growth

**Fig 4:**
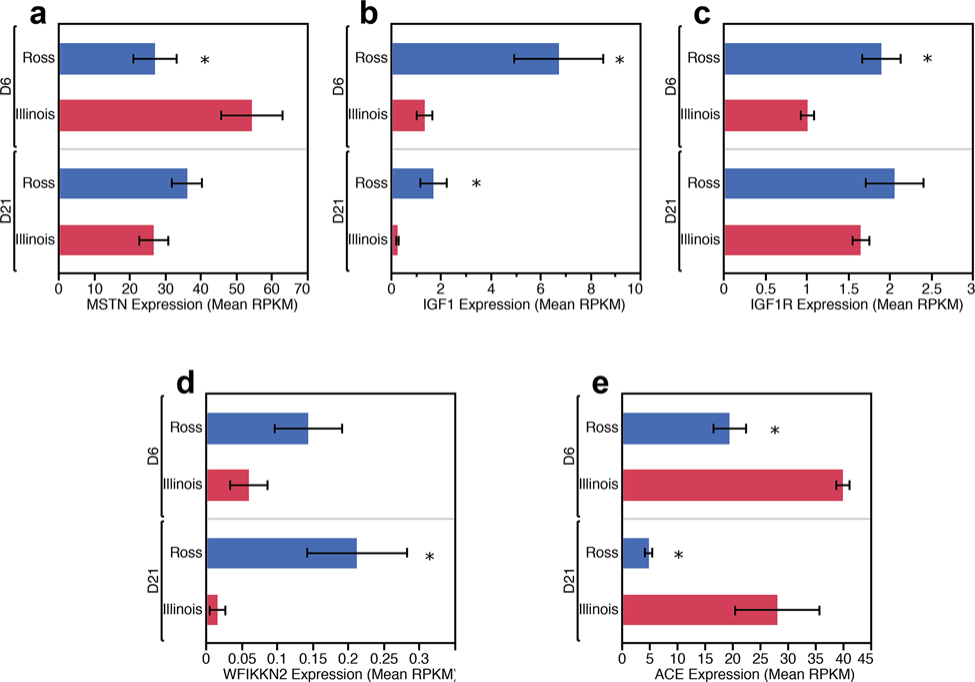
Expression levels, in mean RPKM, of major myogenic growth regulators differentially enriched between Ross 708 and Illinois breast muscle samples. Expression level is the mean RPKM of a given gene for each condition. Error bars represent 1 SEM. Ross 708 D6 n = 6, Illinois D6 n = 6, Ross 708 D21 n = 6, Illinois D21 n = 5. * indicates significant at p < 0.05

**Fig 5:**
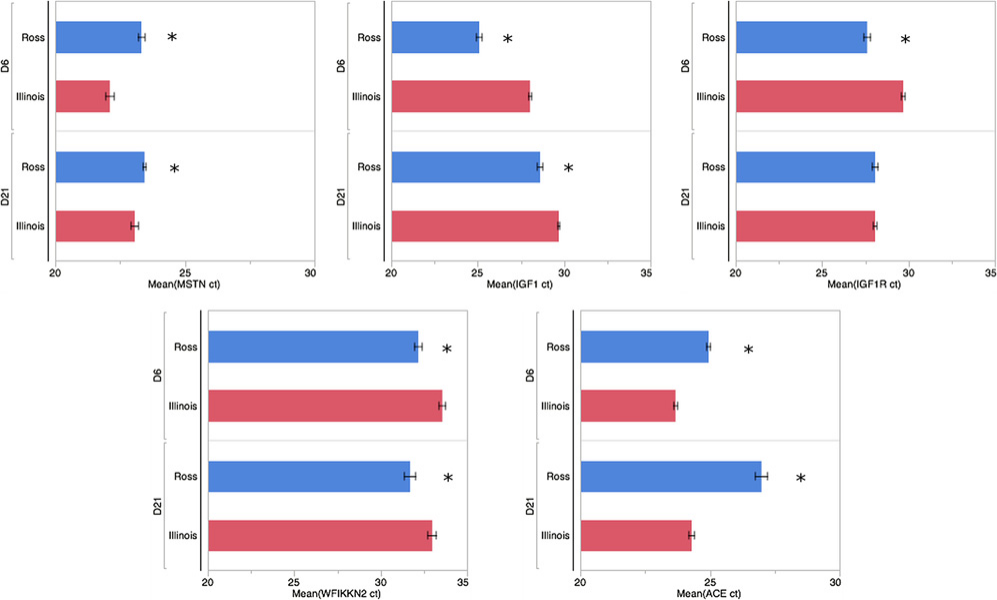
Probe-based qPCR verification of major myogenic growth regulators differentially enriched between Ross 708 and Illinois breast muscle samples. Bars represent the mean ct-value of a given gene for each condition. Error bars represent 1 SEM. Ross 708 D6 n = 6, Illinois D6 n = 5, Ross 708 D21 n = 6, Illinois D21 n = 6. Three Technical replicates of each sample were performed for each gene. * indicates significant at p < 0.05

Four genes that control skeletal muscle growth were differentially regulated between D6 Ross 708 and Illinois breast muscle: *insulin-like growth factor 1* (IGF1), *insulin-like growth factor 1 receptor* (IGF1R), *myostatin* (MSTN), and *angiotensin I converting enzyme 1* (ACE). IGF1 mRNA is enriched in Ross 708 breast muscle compared with the Illinois tissue (Figs 4b and 5b). IGF1 is a positive regulator of proliferation and differentiation in muscle progenitor cells and induces both hyperplastic and hypertrophic growth in muscle [17,18]. IGF1R is the cellular receptor responsible for binding IGF1 and subsequent cellular signaling that promotes hyperplasia and hypertrophy [19] and was also enriched in the Ross 708 samples at the D6 time point (Figs 4c and 5c).

Conversely, two negative regulators of skeletal muscle growth, MSTN (Figs 4a and 5a) and ACE (Figs 4e and 5e), were enriched in the D6 Illinois samples. Loss of function mutations in MSTN, are associated with skeletal muscle hypertrophy in a variety of species including cattle [20,21], sheep [22], mice [23], and humans [24]. *Myostatin* is a TGF-β super-family member and a potent negative regulator of skeletal muscle growth via inhibition of satellite cell proliferation and by altering the protein synthesis/degradation balance of myocytes [18]. Additionally, *myostatin* blocks muscle hypertrophy by inhibiting cell cycle progression and myoblast differentiation [25]. ACE negatively regulates muscle growth by proteolytically converting inactive angiotensin I to the active form, angiotensin II. Angiotensin II increases protein degradation in skeletal muscle through the ubiquitin proteolysis system [26]. Finally, angiotensin II decreases circulating IGF1 levels, potentially further suppressing protein synthesis and skeletal muscle hypertrophy [27].

Several other genes implicated in muscle growth were differentially expressed between the two lines. For example, FOS was enriched in the D6 Ross 708 samples. FOS and JUN (JUN was detected in all conditions but not differentially enriched), form the AP-1 transcription factor complex, which is associated with cell proliferation and differentiation in multiple tissues [28]. FOS has also been identified as an immediate early gene in proliferating satellite cells during human skeletal muscle regeneration [29]. Also enriched in the D6 Ross 708 is the anti-apoptotic factor NR13 which encodes a Bcl-2 family member in the chicken [30]. Addition of *myostatin* to chicken fetal myoblasts results in down-regulation of NR13 [31], suggesting a connection between the regulation of these two genes. In the D6 Ross 708 samples there was enrichment in genes associated with cell cycle and satellite cell proliferation including: *Fanconi anemia complementation group B* (FANCB), *kinesin family member 24* (KIF24), and *nestin* (NES). FANCB encodes a component of the Fanconi E3 ubiquitin ligase complex that plays a critical role in DNA damage, repair and cell cycle progression [32]. KIF24 encodes a centriolar kinesin that localizes to the mother centriole and aids in cell cycle progression [33]. NES is an intermediate filament protein expressed in rapidly dividing progenitor cells of developing and regenerating tissue, and appears to be involved in the rapid assembly and disassembly of structural proteins in dividing cell populations [34]. Finally, Ross 708 D6 muscle was enriched for *musculoskeletal*, *embryonic nuclear protein 1* (MUSTN1), which plays an important role in driving muscle fiber fusion [35].

#### Cellular Metabolism

**Fig 6:**
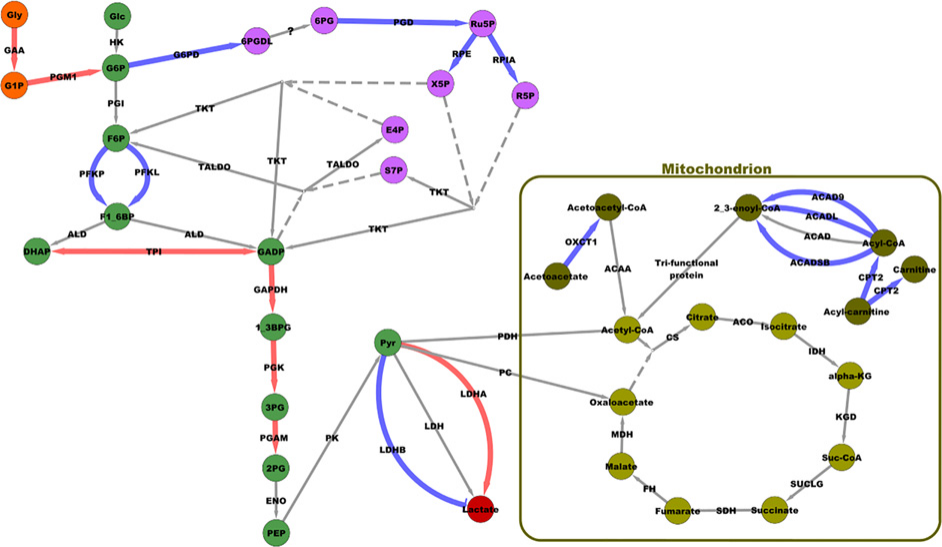
Cellular energy generation network in which several genes identified as differentially enriched between D6 Ross 708 and D6 Illinois are involved. The genes included in the analysis were differentially enriched at the D6 time point between the Ross 708 and Illinois lines. This network includes a number of essential enzymes utilized in cellular metabolism processes including glycolysis, the pentose phosphate pathway and the citric acid cycle. Edges highlighted in red were enriched in D6 Illinois samples; edges highlighted in blue were enriched in D6 Ross 708 samples.

Further comparison of the D6 differentially enriched genes indicates differences in metabolism and energy production between the Illinois and Ross 708 lines (Fig 6). Illinois breast muscle is enriched for genes associated with glycolysis, while Ross 708 muscle is enriched for genes favoring oxidative processes. For example, *protein kinase*, *AMP-activated*, *γ-3* (PRKAG3), which encodes a regulatory subunit of the AMP activated protein kinase (AMPK), was enriched in D6 Illinois samples. AMPK is a master regulator of glucose uptake, glycogen levels, and fatty acid oxidation [36]. Activated AMPK increases glycolytic metabolism while suppressing lipid and cholesterol biosynthesis and metabolism [37]. In addition to PRKAG3, several important glycolytic enzymes are enriched in D6 Illinois samples including: *Triosephosphate isomerase 1* (TPI1), *Glyceraldehyde-3-phosphate dehydrogenase* (GAPDH) and *Phosphoglycerate kinase 1* (PGK1). TPI1 encodes the subunits of a homodimeric enzyme that catalyzes the isomerization of dihydroxy-acetone phosphate (DHAP) to glyceraldehyde-3-phosphate (GADP). The next step in the glycolysis is catalyzed by GAPDH, which catalyzes the phosphorylation of GADP to 1,3-bisphosphoglycerate (1,3BPG). Finally, *Phosphoglycerate Kinase 1* (PGK1) catalyzes the conversion of 1,3BPG to 3-phosphoglycerate (3-PG), yielding 2 ATP in the process.

Complementing enrichment of transcripts encoding glycolytic enzymes, in the D6 Illinois birds is increased expression of two genes, *α-acid glucosidase* (GAA) and *phosphoglucomutase 1* (PGM1), that connect glycogen breakdown to glycolysis. GAA is a lysosomal enzyme essential for the release of glucose from glycogen while PGM1 reversibly catalyzes the isomerization of glucose-1-phosphate, the result of glycogen breakdown, to glucose-6-phosphate, which is the entry point for glycolysis. In addition, *lactate dehydrogenase A* (LDHA), which represents a branching point in the production of energy from glucose, is enriched in D6 Illinois samples. Pyruvate can either enter the oxidative TCA cycle or be converted to lactate by LDH for export from the cell [38]. LDH is a tetrameric enzyme composed of various combinations of two subunits, LDHA and LDHB. The enzymatic activity of these subunits varies with LDHB exhibiting maximum activity at low pyruvate concentrations while LDHA maintains activity at high pyruvate concentrations. The ability of LDHA to maintain catalytic function in high concentrations of pyruvate favors the conversion of pyruvate to lactate and, thus, a shift away from oxidative metabolism [38]. This is consistent with the fast-muscle type seen in the breast muscle of chickens [39].

Further analysis of the D6 Ross 708 breast muscle samples suggest that this tissue is enriched for gene products supporting energy production via oxidative phosphorylation. *Peroxisome proliferator-activated receptor γ co-activator 1-α* (PPARGC1A) is a transcriptional co-activator enriched in the D6 Ross 708 samples that promotes myoblast proliferation, mitochondrial biogenesis, and lipid transport, along with fatty acid and glucose oxidation [40–42]. Six gene transcripts that function in acyl oxidation are enriched in the D6 Ross 708 muscle: CD36, *acyl-CoA dehydrogenase 9* (ACAD9), *acyl-CoA dehydrogenase*, *long chain* (ACADL) and *acyl-CoA dehydrogenase short/branched chain* (ACADSB), *3-oxoacid CoA transferase 1*(OXCT1), and *carnitine palmitoyltransferase 2* (CPT2). CD36 is a class B scavenger receptor that functions as a fatty acid transporter in chicken skeletal muscle [43]. CD36 localizes to the plasma membrane and mitochondria; at the plasma membrane CD36 functions in fatty acid uptake, whereas in the mitochondria it functions to import fatty acids for oxidation [43]. ACAD9, ACADL and ACADSB, all function in the oxidation of their respective acyl substrates while OXCT1 functions in ketone body oxidation. Enrichment for CPT2 in the D6 Ross 708 breast muscle likely increases the flow of acyl chains into the mitochondria for coupling to coenzyme A. Finally, one member of the TCA Cycle, *succinate-CoA-ligase*, *GDP forming beta subunit* (SUCLG2) is also enriched in the D6 Ross 708 breast muscle. This subunit favors formation of GTP instead of ATP as the acyl-CoA products traverse the cycle [44,45].

Ross D6 breast muscle is also enriched in four transcripts encoding proteins that function in the pentose phosphate pathway (PPP): *hexose-6-phosphate dehydrogenase* (H6PD), *phosphogluconate dehydrogenase* (PGD), *ribulose-5-phosphate-3 epimerase* (RPE), and *transketolase* (TKT). The PPP is responsible for production of NADPH, which provides reducing equivalents essential to anabolism [46]. H6PD catalyzes the first, rate-limiting, step in this pathway by catalyzing the oxidation of glucose-6-phosphate to 6-phosphogluconolactone with the production of one molecule of NADPH per reaction. PGD catalyzes the second NADPH producing step while RPE and TKT function in non-oxidative steps of the PPP. In addition to these members of the PPP, Ross 708 D6 breast muscle is also enriched for cytoplasmic *isocitrate Dehydrogenase 1* (IDH1). IDH uses NADP to oxidize isocitrate ultimately to alpha-ketoglutarate, yielding a molecule of NADPH.

#### Innervation

In addition to detecting differentially expressed genes affecting muscle growth and metabolism, several genes affecting innervation and neuromuscular junctions were significantly different between the Ross 708 and Illinois birds at day 6. Formation of the neuromuscular junction (NMJ) is a complex process requiring temporally and spatially coordinated interactions between nerve terminals and muscles. Branching growth and development of the nervous system and interaction with the muscle triggers the formation of the NMJ, however innervation is not necessary for skeletal muscle growth [47]. Several genes involved in innervation and neuromuscular junction formation were enriched in the D6 Illinois samples compared with the Ross 708 breast muscle. Among these was *purinergic receptor P2Y*, *G-protein coupled*, *1* (P2RY1), which localizes to the neuromuscular junction [48], and acts as a trophic factor to promote localization of acetylcholine receptor and acetylcholine esterase to the NMJ [48]. Additionally, the *potassium inward-rectifying channel*, *subfamily J*, *member 12* (KCNJ12,), which localizes to the NMJ [49] was enriched in D6 Illinois samples. The *kyphoscoliosis peptidase* (KY) transcript, also enriched in D6 Illinois, encodes a cytoskeleton-associated protease that is involved in proper maturation and stabilization of NMJs [50].

The D6 Illinois samples also showed enrichment for neuronal specific genes involved in NMJ formation including *vesicle-associate membrane protein 1* (VAMP1), *neurogranin* (NRGN), and *leucine-rich repeat and Ig domain containing 1* (LINGO1). VAMP1 is a primary component of the protein complex involved in docking and/or fusion of synaptic vesicles with the presynaptic membrane [51]. VAMP1 is not found in satellite cells or myofibers, but rather in pre-synaptic regions of neurons in muscle [52]. NRGN is a post-synaptic protein kinase substrate that is involved in calmodulin signaling, and acts as a third messenger in synaptic development and remodeling [53]. LINGO1 encodes a component of the Nogo-A signaling receptor complex. Nogo-A signaling affects the cytoskeleton of neurons through RhoA and Rho Kinase (ROCK) activity and is critical for branching of peripheral nerves [54]. Two genes products potentially involved in innervation were enriched in the D6 Ross 708 samples: *cholinergic receptor*, *nicotinic*, *alpha 1* (CHRNA1) and *cholinergic receptor*, *muscarinic 4 CHRM4*. Both of these receptor subunits are found at the neuromuscular junctions [55].

### Day 21 Differentially Enriched Genes

#### Muscle Growth

In the D21 samples expression levels of IGF1 remained enriched in Ross 708 (Figs 4b and 5b) samples while ACE transcripts remained elevated in the Illinois breast muscle (Figs 4e and 5e). In contrast, by post-hatch day 21 the Ross 708 and the Illinois samples showed similar expression of IGF1R (Fig 4c) and MSTN (Fig 4a) in the RNA-seq data. Probe-based qPCR confirmed similar expression of IGF1R between Ross 708 and Illinois samples (Fig 5c); however probe-based qPCR results indicated that MSTN mRNA expression remains significantly enriched in the Illinois samples at the D21 time point (Fig 5a). In addition to these factors, the *WAP*, *follistatin/kazal*, *immunoglobulin*, *kunitz and netrin domain containing 2* (WFIKKN2) gene was enriched in D21 Ross 708 breast muscle (Fig 4d). WFIKKN2 is a negative regulator of MSTN through its follistatin domain, and overexpression of WFIKKN2 in mice causes muscle hypertrophy after birth by sequestering MSTN [56–58]. Unlike the D21 samples, the RNA-seq expression data did not show a significant enrichment for WFIKKN2 mRNA at the D6 time point (Fig 4d); however probe-based qPCR follow-up showed both D6 and D21 Ross 708 samples to be significantly enriched for WFIKKN2 mRNA (Fig 5d).

Another D21 Ross 708 enriched transcript with a role in myogenesis encodes *small muscle protein*, *x-linked* (SMPX). Myoblasts over-expressing SMPX are enlarged due to formation of membrane ruffles and lamellipodia and IGF1 supplementation of cultures overexpressing SMPX induces myotube hypertrophy [59]. The *HOP homeobox* gene (HOPX) mRNA is enriched in D21 Ross breast muscle. HOPX encodes an atypical homeobox protein that does not have the capacity to bind DNA but can potentiate skeletal muscle differentiation through interaction with enhancer of polycomb 1 (Epc1) [60]. Epc1 was expressed in all conditions but was not differentially expressed. In mice, HOPX knockout individuals showed an inhibition of skeletal muscle differentiation and impairments in muscle regeneration [60].

*Cysteine and glycine-rich protein 3* (CSRP3), which is associated with myogenic differentiation was also enriched in D21 Ross 708 breast muscle relative to D21 Illinois samples. CSRP3 is found in both the cytosol and the nucleus; the cytosolic form serves as a scaffold for structural proteins, while the nuclear CSRP3 serves as a cofactor for basic helix-loop-helix (bHLH) transcription factors including MYOD1 and myogenin (detected in both lines on both days). These transcription factors bind the E-box motif and promote the transcription of genes at multiple critical stages of muscle growth and differentiation [61,62]. In the Illinois line, only one additional gene affecting muscle growth, MYOD1, was enriched at D21. MYOD1 is responsible for withdrawal from the cell cycle and commitment toward terminal myogenic differentiation [63].

#### Cellular Metabolism and Innervation

As at D6, IDH1 is still elevated in the D21 Ross 708 compared with the D21 Illinois breast muscle. The D21 Ross 708 breast muscle shows enrichment for *midikine* (MDK), which encodes a small neurite-derived heparin binding growth factor that participates in signaling during NMJ formation [64]

### Implications of Day 6 and 21 Gene Expression Patterns

**Fig 7:**
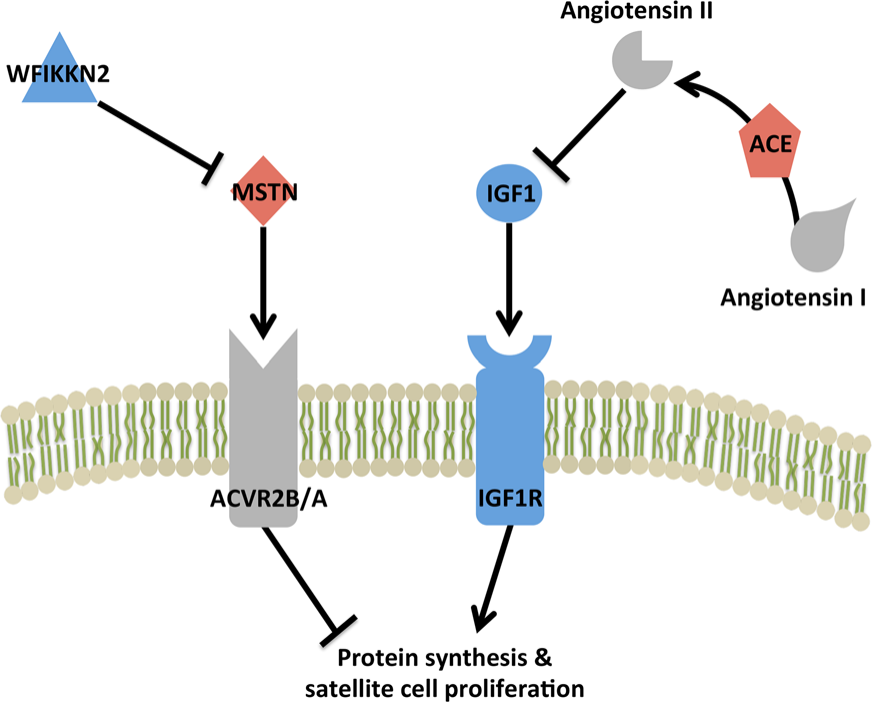
Summary of the interactions between major growth factors, differentially expressed between the Ross 708 and Illinois breast muscle samples, and their affect on myogenic growth. Genes highlighted orange were enriched in Illinois samples, genes highlighted in blue were enriched in Ross 708 samples.

Myogenesis is a complex process that begins with a pool of proliferative progenitor cells and results in terminally differentiated, multi-nucleated myofibers. Typically the number of muscle fibers is determined during the embryonic stage of development [65]. In the post-hatch chicken, the increase in muscle size is a result of proliferation and fusion of myogenic progenitor cells (satellite cells) to existing myotubes, increasing the number of nuclei and the capacity for protein synthesis [66]. Furthermore, breast muscle growth in these broiler chicken lines can be divided into two time frames: pre and post-day 14 [8]. From hatch through day 14, the breast muscle of both the modern Ross 708 and Illinois line exhibit rapid growth relative to the total body, although the Ross 708 breast muscle grows approximately 3-fold faster than the Illinois line during this time. When normalized to the total bird mass, the relative growth of the Illinois breast muscle plateaus after post-hatch day 14, while the Ross 708 birds continues to increase the relative proportion of breast muscle. While the breast muscle continues to grow in both lines, only the Ross 708 birds continue to add more breast muscle relative to the growth of other tissues after D14 [8]. We hypothesize that IGF1, IGF1R, MSTN, WFIKKN2 and ACE may form the basis for a network of interacting factors in which the breast muscle growth is a consequence of the ratio of these growth promoting and inhibiting factors (Fig 7). The significantly elevated levels of IGF1, IGF1R and WFIKKN2 compared to MSTN and ACE seen at day 6 (Figs 4 and 5) in the Ross 708 birds may play a role the rapid relative growth of this breast muscle tissue prior to day 14. By day 21 post-hatch, the ratio of IGF, IGF1R and WFIKKN2 to MSTN and ACE has decreased, but is still higher in the Ross 708 tissue relative to Illinois breast muscle. Conceivably, this elevated ratio of growth promoters to inhibitors results in the positive allometric growth of the Ross 708 breast muscle while the growth of this muscle in the Illinois birds becomes proportional to other tissues. Given the pleiotropic effects of IGF, MSTN [67] and ACE, selection that affects changes in their expression would be expected to have multiple, systemic effects on organ systems.

## Conclusions

We have compared gene expression patterns between the breast muscle tissues of an Illinois and modern chicken line. The Illinois line had been selected for improved growth performance until the late 1940s while the Ross 708 line is still undergoing selection for meat production traits. A major distinction between these lines is the significant elevation in breast muscle yield in the modern (Ross 708) line. We hypothesized that selection has affected expression of multiple genes affecting muscle growth. Based on this transcriptome analysis, likely gene candidates for direct effect on muscle yield include IGF1, IGF1R, WFIKKN2, MSTN and ACE. Furthermore, these growth factors may affect other allometric differences between the Illinois and Ross 708 lines [8]. IGF1 has been shown to affect allometric growth in several species [68–70]. However, it is unlikely that all morphometric differences between the lines are due to allelic variations in genes that are transcribed and translated. For example, non-coding RNAs are differentially expressed between lines selected for divergent skeletal muscle growth [71]. Also, changes in epigenetic regulation may have been driven by the need to support rapid growth of modern broilers. Future studies will explore both non-coding and epigenetic differences between these legacy and modern chicken lines.

## Supporting Information

S1 Table A table of all genes determined to be differentially enriched in either the Ross 708 or Illinois line, at the D6 time point. The table columns (in order) are the Entrez IDs, gene symbol, the line in which the gene is enriched, the log_2_ of the ratio of the Ross 708 RPKM to the Illinois RPKM, the mean RPKM for the Ross 708 samples, the mean RPKM for the Illinois samples, and the individual RPKM values for each library analyzed.
(XLSX)

S2 Table A table of all genes determined to be differentially enriched in either the Ross 708 or Illinois line, at the D21 time point. The table columns (in order) are the Entrez IDs, gene symbol, the line in which the gene is enriched, the log_2_ of the ratio of the Ross 708 RPKM to the Illinois RPKM, the mean RPKM for the Ross 708 samples, the mean RPKM for the Illinois samples, and the individual expression values (in RPKM) for each library analyzed.
(XLSX)
